# Supplementary material for: Re-expression of CA1 and entorhinal activity patterns preserves temporal context memory at long timescales
Source: Nat Commun. 2023 Jul 19;14:4350. doi: 10.1038/s41467-023-40100-8 (PMC10356845; doi:10.1038/s41467-023-40100-8)
Supplement: Supplementary file 1 — Supplementary Information [file 41467_2023_40100_MOESM1_ESM.pdf]

SUPPLEMENTARY INFORMATION

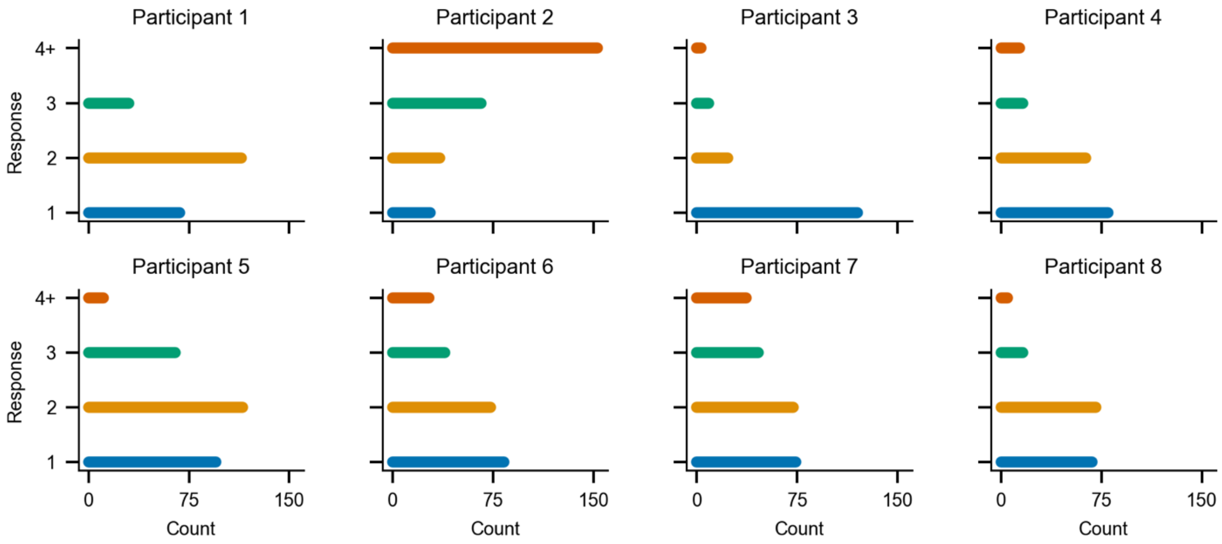

Supplementary Figure 1. Subjective estimates of image frequency in the final memory phase. During the frequency test, participants were asked to indicate how many times they had seen each image (1, 2, 3, or 4 or more times). No participant preferentially chose the correct response (i.e., three times). Source data are provided as a Source Data file.

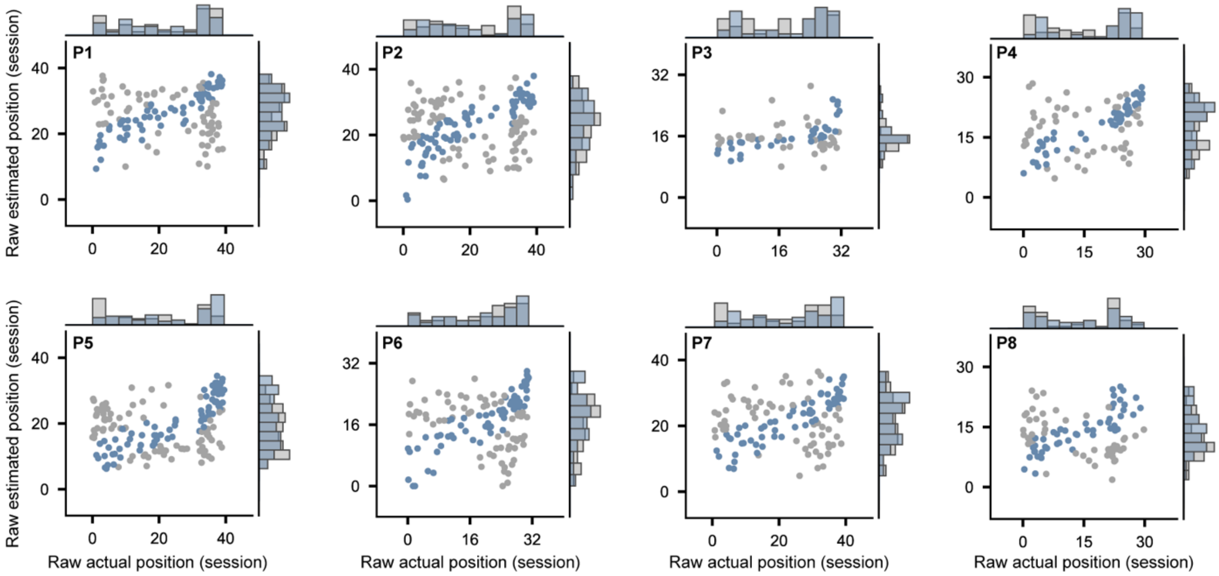

Supplementary Figure 2. Raw estimated and actual temporal positions for each participant. Each dot indicates a temporal memory judgment. Temporal judgments for each participant were divided into 'high temporal precision' and 'low temporal precision' on the basis of temporal memory error (median split). Histograms on sides indicate distributions of raw estimated (vertical) and actual (horizontal) temporal positions (overlaid across high- and low- temporal precision). Colors denote temporal memory precision (blue: high temporal precision; gray: low temporal precision). Source data are provided as a Source Data file.

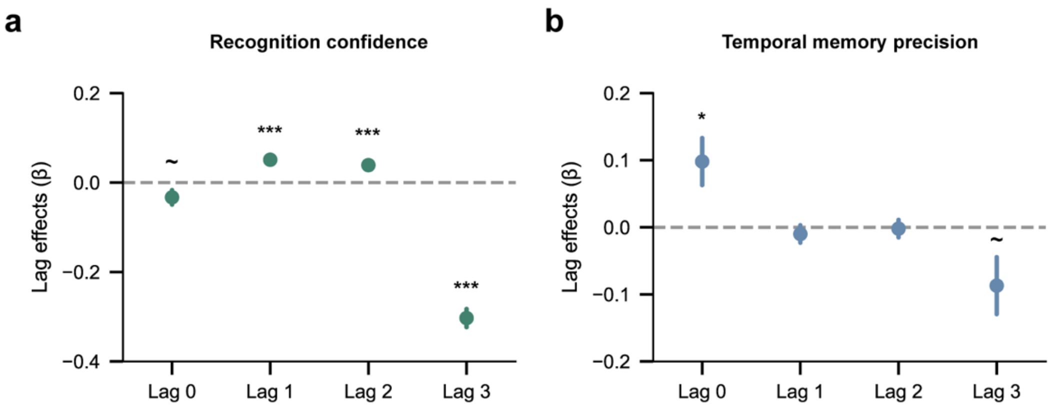

Supplementary Figure 3. Lag effects on behavioral measures of final memory. (a) Lag effects on recognition confidence. There were spacing effects in recognition confidence such that confidence increased as a function of increasing temporal lags between exposures (lag 1: $\beta = 0.051$, $p < 0.001$, 95% CI = [0.04, 0.07]; lag 2: $\beta = 0.039$, p < 0.001, 95% CI = [0.02, 0.05]; in a mixed-effects linear regression model with recognition confidence as dependent variable and with each temporal lag as a separate predictor, $n = 8$ independent participants), and a forgetting effect wherein recognition confidence diminished as the lag between the last exposure and the final memory test increased (lag 3: $\beta = -0.303$, $p < 0.001$, 95% CI = [-0.35, -0.25]). **(b)** Lag effects on temporal memory precision. There was a recency effect across months with temporal memory precision increasing the later in the experiment an image was first encountered (lag 0: $\beta = 0.098$, $p = 0.021$, 95% CI = [0.02, 0.18]; in a mixed-effects logistic regression model with temporal memory precision as dependent variable and with each temporal lag as a separate predictor, $n = 8$ independent participants). Error bars reflect mean ± s.e.m.; ~$p < 0.10$; *$p < 0.05$; ***$p < 0.001$. Source data are provided as a Source Data file.

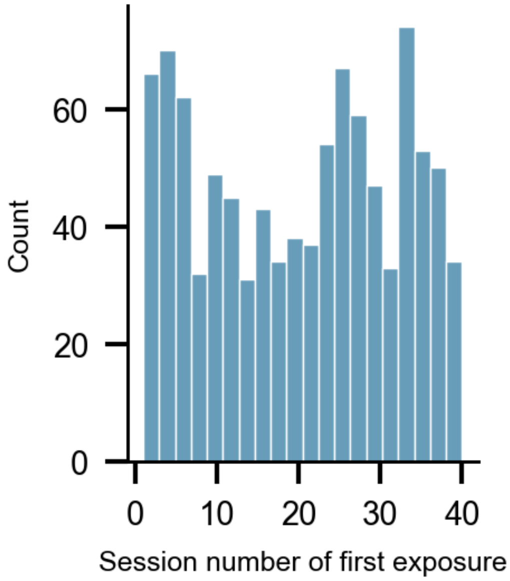

Supplementary Figure 4. Distribution of images' first exposures. Counts of first exposures (E1) for old images included in analyses as a function of session number. Note: counts are summed across participants. Source data are provided as a Source Data file.

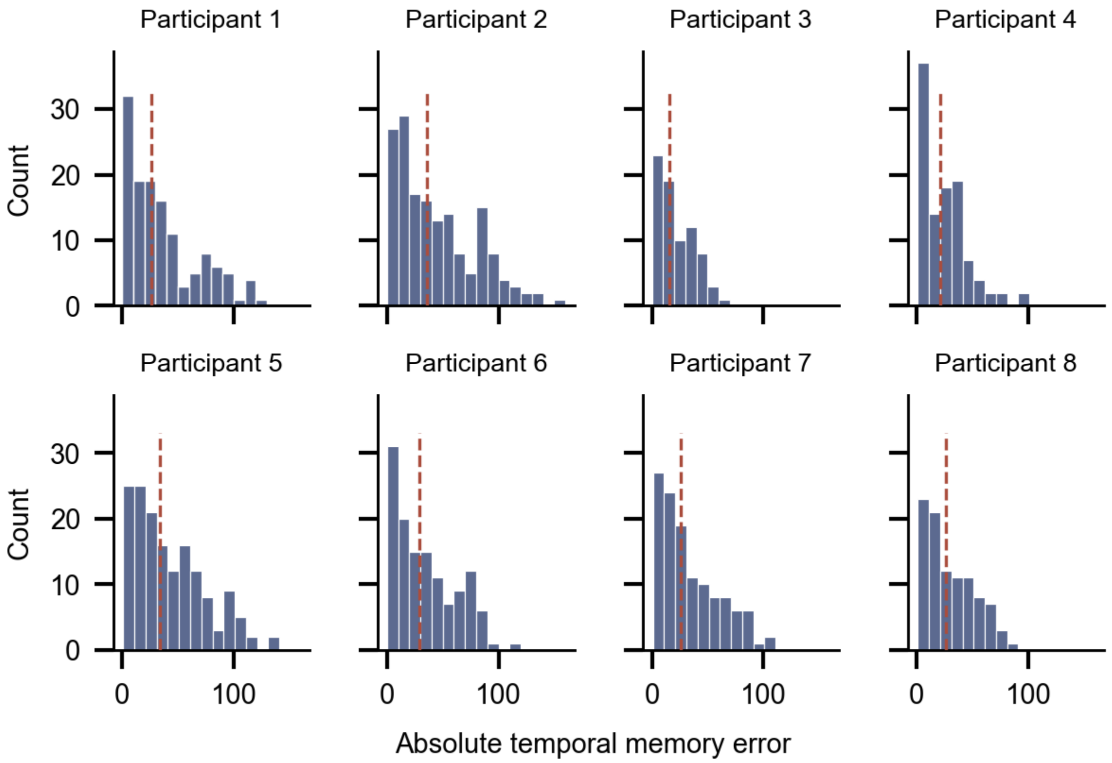

Supplementary Figure 5. Distribution of individual participant's temporal memory error. Item-wise temporal memory error was calculated using the absolute difference between the ranked estimated temporal position and the ranked actual position. Red line denotes median value of the absolute temporal error. As the resulting distribution was highly non-normal, a median split was performed for all subsequent analyses of temporal error as a factor. Source data are provided as a Source Data file.

Cortical ROIs (HCP-MMP1 atlas)	*z*-value	*p*-value (uncorrected)
L Frontal Eye Fields	2.027	0.043
L Supplementary and Cingulate Eye Field	2.693	0.007
L Area 6m anterior	2.761	0.006
L Area IFJp	2.356	0.018
L Area IFSp	2.043	0.041
L Area posterior 9-46v	2.020	0.043
L Entorhinal Cortex	2.001	0.045
L Perirhinal Ectorhinal Cortex	2.912	0.004
L Area 31pd	-2.011	0.044
R Area 31p ventral	-2.369	0.018
R Frontal Opercular Area 1	-2.062	0.039
R Entorhinal Cortex	2.082	0.037
R Area TG dorsal	2.109	0.035
R AreaTemporoParietoOcci pital Junction 1	2.119	0.034
R Area 31a	-1.963	0.050

Supplementary Table 1. Whole-brain representational similarity analysis in cortical ROIs. A mixed-effects logistic regression predicting temporal memory with pattern similarity as the main fixed effect of interest was conducted for each parcel of the HCP-MMP1 atlas to determine whether cortical regions outside of the MTL also exhibit pattern similarity effects on temporal memory. This table summarizes the whole-brain parcel level analysis with cortical ROIs showing significant relationships between pattern similarity and temporal memory before correcting for multiple comparisons (*p(uncorrected)* < 0.05). No regions survived correction for multiple comparisons.
