# Supplementary material for: Re-expression of CA1 and entorhinal activity patterns preserves temporal context memory at long timescales
Source: Nat Commun. 2023 Jul 19;14:4350. doi: 10.1038/s41467-023-40100-8 (PMC10356845; doi:10.1038/s41467-023-40100-8)
Supplement: Supplementary file 3 — Description of Additional Supplementary Files [file 41467_2023_40100_MOESM3_ESM.pdf]

Description of Additional Supplementary Files:

Supplementary Movie 1: Example trials of the final memory test.
