# Supplementary material for: Re-expression of CA1 and entorhinal activity patterns preserves temporal context memory at long timescales
Source: Nat Commun. 2023 Jul 19;14:4350. doi: 10.1038/s41467-023-40100-8 (PMC10356845; doi:10.1038/s41467-023-40100-8)
Supplement: Supplementary file 5 — Reporting Summary [file 41467_2023_40100_MOESM5_ESM.pdf]

nature portfolio

Corresponding author(s): Futing Zou

Last updated by author(s): May 19, 2023

Reporting Summary

Nature Portfolio wishes to improve the reproducibility of the work that we publish. This form provides structure for consistency and transparency in reporting. For further information on Nature Portfolio policies, see our Editorial Policies and the Editorial Policy Checklist.

Statistics

For all statistical analyses, confirm that the following items are present in the figure legend, table legend, main text, or Methods section.

n/a	Confirmed	
☐	☒	The exact sample size (*n*) for each experimental group/condition, given as a discrete number and unit of measurement
☐	☒	A statement on whether measurements were taken from distinct samples or whether the same sample was measured repeatedly
☐	☒	The statistical test(s) used AND whether they are one- or two-sided<br>*Only common tests should be described solely by name; describe more complex techniques in the Methods section.*
☐	☒	A description of all covariates tested
☐	☒	A description of any assumptions or corrections, such as tests of normality and adjustment for multiple comparisons
☐	☒	A full description of the statistical parameters including central tendency (e.g. means) or other basic estimates (e.g. regression coefficient) AND variation (e.g. standard deviation) or associated estimates of uncertainty (e.g. confidence intervals)
☐	☒	For null hypothesis testing, the test statistic (e.g. *F*, *t*, *r*) with confidence intervals, effect sizes, degrees of freedom and *P* value noted<br>*Give P values as exact values whenever suitable.*
☒	☐	For Bayesian analysis, information on the choice of priors and Markov chain Monte Carlo settings
☒	☐	For hierarchical and complex designs, identification of the appropriate level for tests and full reporting of outcomes
☐	☒	Estimates of effect sizes (e.g. Cohen's *d*, Pearson's *r*), indicating how they were calculated

Our web collection on statistics for biologists contains articles on many of the points above.

Software and code

Policy information about availability of computer code

Data collection	We used Psychophysics Toolbox 3.0.14 and MATLAB R2018a for stimulus presentation and response collection.
Data analysis	We used custom Python 3 scripts for all analyses in this paper.

For manuscripts utilizing custom algorithms or software that are central to the research but not yet described in published literature, software must be made available to editors and reviewers. We strongly encourage code deposition in a community repository (e.g. GitHub). See the Nature Portfolio guidelines for submitting code & software for further information.

Data

Policy information about availability of data

All manuscripts must include a data availability statement. This statement should provide the following information, where applicable:

- Accession codes, unique identifiers, or web links for publicly available datasets
- A description of any restrictions on data availability
- For clinical datasets or third party data, please ensure that the statement adheres to our policy

The NSD dataset is freely available at http://naturalscenesdataset.org (Allen et al., 2021). Images used for this paper were taken from the Common Objects in Context database (Lin et al., 2014) (https://cocodataset.org). Source data are provided with this paper.

Human research participants

Policy information about studies involving human research participants and Sex and Gender in Research.

Reporting on sex and gender	A mixture of males and females were used (2M, 6F). Biological sex/gender was self-reported by participants. Sex and gender were not included as covariates in the analyses because we did not have any a priori hypotheses or research questions about this. Neither sex or gender is likely to influence our findings.
Population characteristics	Human participants included 2 males and 6 females. Subjects were healthy young adults between 19–32 years old at the time of participation. All participants had normal or corrected-to-normal visual acuity, normal color vision, and no MRI contraindications.
Recruitment	Participants were recruited through advertisements to the local community and were screened based on ability to participate in this long-term neuroimaging study. In addition, we selected participants based on data quality from an initial 7T fMRI session. This selection does induce a bias towards individuals with low head motion, high cognitive performance, and strong BOLD responses. The goal of the selection is to optimize the quality of the NSD dataset, and does not represent an unbiased sampling of the human population. The self-selection is unlikely to influence our findings.
Ethics oversight	University of Minnesota Institutional Review Board

Note that full information on the approval of the study protocol must also be provided in the manuscript.

Field-specific reporting

Please select the one below that is the best fit for your research. If you are not sure, read the appropriate sections before making your selection.

[x] Life sciences [ ] Behavioural & social sciences [ ] Ecological, evolutionary & environmental sciences

For a reference copy of the document with all sections, see nature.com/documents/nr-reporting-summary-flat.pdf

Life sciences study design

All studies must disclose on these points even when the disclosure is negative.

Sample size	This study collects massive amounts of data in individual subjects. Analyses demonstrated in this paper are conducted at both group-level and within-subject level, demonstrating the precision and robustness of the data collected. For group-level analyses, the number of subjects used for NSD (n = 8) is sufficiently large to provide some power for statistical inference. The sample size (n = 8) was chosen based on consideration of guarding against incidental findings that occur only in a few individuals and based on consideration of subject burden (if all images had been presented to a single subject, data collection would have extended for 8 years).
Data exclusions	We implemented a subject-selection procedure in which the best 8 subjects out of a pool of 14 potential subjects (on basis of criteria such as head motion and BOLD signal strength) were selected for full NSD data acquisition. We used the entire collected data in our analyses.
Replication	This original resource paper of the dataset (Allen et al., 2021) described extensive quality checks on the data acquired from the 8 NSD subjects. We provided substantial evidence that high-quality data were obtained from all subjects. A replication experiment was not performed in this study.
Randomization	All participants engaged in the same set of experiments. However, somewhat non-overlapping sets of stimuli were chosen for each subject. The allocation of stimuli to different subjects during the continuous recognition phase was done randomly from a fixed set of images pulled from the Microsoft COCO database. Given the large scale of stimulus sampling (e.g. 9,000–10,000 unique images were shown to each subject), it is likely that although the exact same images are not shown to each subject, the same general types of stimulus features are well sampled for each subject. Approximately half of the old images used in the final memory test were semi-randomly selected based on their presentation time in the continuous recognition phase. Another half were semi-randomly selected to maximally span semantic space.
Blinding	Blinding is not relevant to this study given that there is little that the investigators could have done to bias the nature of the recorded data and given that the participants do not belong to any discrete groupings.

Reporting for specific materials, systems and methods

We require information from authors about some types of materials, experimental systems and methods used in many studies. Here, indicate whether each material, system or method listed is relevant to your study. If you are not sure if a list item applies to your research, read the appropriate section before selecting a response.

Materials & experimental systems

n/a	Involved in the study	
☒	☐	Antibodies
☒	☐	Eukaryotic cell lines
☒	☐	Palaeontology and archaeology
☒	☐	Animals and other organisms
☒	☐	Clinical data
☒	☐	Dual use research of concern

Methods

n/a	Involved in the study	
☒	☐	ChIP-seq
☒	☐	Flow cytometry
☐	☒	MRI-based neuroimaging

Magnetic resonance imaging

Experimental design

Design type	The NSD continuous recognition experiment is task-based and has an event-related design.
Design specifications	In the continuous recognition experiment, images were presented for 3 seconds, and were followed by a minimum of 1 second of gap before the next trial. Many thousands of distinct images were presented over the course of many distinct scan sessions, with a maximum number of presentations per distinct image of 3.
Behavioral performance measures	Button presses and associated reaction times for each trial in the continuous recognition experiment were recorded. We quantified recognition performance using signal detection theory.

Acquisition

Imaging type(s)	Functional and structural
Field strength	7T and 3T
Sequence & imaging parameters	The primary fMRI sequence involved gradient-echo EPI, FOV 216 mm x 216 mm, matrix size 120 x 120, slice thickness 1.8 mm, orientation axial, TR 1.6 s, TE 22.0 ms, and flip angle 62°.
Area of acquisition	Whole-brain scans

Diffusion MRI ☐ Used ☒ Not used

Preprocessing

Preprocessing software	A combination of custom MATLAB and Python code, FreeSurfer 6, and selected tools from SPM, FSL, ANTs, and MRTrix3.
Normalization	For volume analysis, fMRI data used in this paper were prepared in subject-native spaces. For surface analysis, fMRI data used in this paper were prepared in atlas spaces.
Normalization template	Fsaverage templates were used for preparation of data in atlas spaces.
Noise and artifact removal	We used the beta version 2 of the NSD dataset (Allen et al., 2021). For the GLM preparation of the data, we used a library of hemodynamic response functions (HRFs) derived from an initial analysis of the dataset as an efficient and well-regularized method for estimating voxel-specific HRFs.
Volume censoring	No censoring was performed.

Statistical modeling & inference

Model type and settings	Trial-wise fMRI response amplitudes were estimated for individual voxels in individual subjects. Representational similarity analyses were then performed on these response amplitudes.
Effect(s) tested	We conducted rich sampling of the brain's response to a large number of complex natural scenes in the continuous recognition experiment and a final memory experiment that separately measured recognition and temporal memory. The resulting measurements was then used to test whether neural pattern similarity across repeated image exposures predicted subsequent temporal memory precision. The relationship between pattern similarity and temporal memory was tested using mixed-effects logistic regression models for each ROI. We also used permutation tests to test whether pattern similarity within an ROI was greater for high temporal memory precision than low temporal memory precision.

Specify type of analysis: ☐ Whole brain ☒ ROI-based ☐ Both

Anatomical location(s)	Manually defined regions of interest in the medial temporal lobe (MTL) were created following a 7T protocol for segmentation of MTL subregions (Berron et al., 2017). Early visual cortex ROI (V1) was manually drawn based on results of a population receptive field experiment from the NSD reported in the original resource paper of the data (Allen et al., 2021). Atlas-based regions of interest were determined based on a cortical parcellation atlas (Glasser et al., 2016).

Statistic type for inference (See Eklund et al. 2016)	Permutation-based significance tests and mixed-effects models.
Correction	For the representational similarity analysis, we applied Holm-Bonferroni corrections for multiple comparisons across MTL ROIs or cortical parcels.

Models & analysis

n/a	Involved in the study
☒	☐ Functional and/or effective connectivity
☒	☐ Graph analysis
☒	☐ Multivariate modeling or predictive analysis
